# Current Understanding of Leaf Senescence in Rice

**DOI:** 10.3390/ijms22094515

**Published:** 2021-04-26

**Authors:** Sichul Lee, Celine Masclaux-Daubresse

**Affiliations:** 1 Center for Plant Aging Research, Institute for Basic Science (IBS), Daegu 42988, Korea; 2 Institut Jean-Pierre Bourgin, INRAE, AgroParisTech, Université Paris-Saclay, 78000 Versailles, France; celine.masclaux-daubresse@inrae.fr

**Keywords:** rice, leaf senescence, productivity, chlorophyll breakdown, stay-green, transcription factors, phytohormones, nitrogen remobilization

## Abstract

Leaf senescence, which is the last developmental phase of plant growth, is controlled by multiple genetic and environmental factors. Leaf yellowing is a visual indicator of senescence due to the loss of the green pigment chlorophyll. During senescence, the methodical disassembly of macromolecules occurs, facilitating nutrient recycling and translocation from the sink to the source organs, which is critical for plant fitness and productivity. Leaf senescence is a complex and tightly regulated process, with coordinated actions of multiple pathways, responding to a sophisticated integration of leaf age and various environmental signals. Many studies have been carried out to understand the leaf senescence-associated molecular mechanisms including the chlorophyll breakdown, phytohormonal and transcriptional regulation, interaction with environmental signals, and associated metabolic changes. The metabolic reprogramming and nutrient recycling occurring during leaf senescence highlight the fundamental role of this developmental stage for the nutrient economy at the whole plant level. The strong impact of the senescence-associated nutrient remobilization on cereal productivity and grain quality is of interest in many breeding programs. This review summarizes our current knowledge in rice on (i) the actors of chlorophyll degradation, (ii) the identification of stay-green genotypes, (iii) the identification of transcription factors involved in the regulation of leaf senescence, (iv) the roles of leaf-senescence-associated nitrogen enzymes on plant performance, and (v) stress-induced senescence. Compiling the different advances obtained on rice leaf senescence will provide a framework for future rice breeding strategies to improve grain yield.

## 1. Introduction

The leaf is the primary photosynthetic organ for energy production and nutrient assimilation at the growth and mature development stages [1]. Senescence occurs in a coordinated manner, starting from the tip and margins of the leaf toward the base and petiole in many plant species [2]. In these tissues, cells undergo a sequential disorganization of cellular organelles, and changes in gene expression and metabolism are orderly managed [1,2,3]. Leaf senescence is the last step of organ life and the last development stage in their life history for many plant species. Senescence events are basically controlled by age information and occur irrespective of environmental conditions. However, leaf senescence onset and senescence intensity can be modulated by many internal cues that perceive and integrate phytohormonal signals, nutrient status, water usage, light quality and length, and climate change [1]. Although leaf senescence is mainly described by the visual yellowing phenotype related to chlorophyll breakdown, as it can be seen by autumnal color changes of leaves, chlorophyll degradation is not the first event in the long leaf senescence program [4].

Basically, plants follow monocarpic senescence or polycarpic senescence [5,6]. Monocarpic plants such as annual cereal crops have only a single reproductive event in their life cycle. They produce a high number of mature offspring, such as seeds, and will initiate the next life cycle upon germination [5,6]. On the other hand, polycarpic plants (also known as perennial plants) such as trees and shrubs undergo many reproduction cycles and continue growing across many seasons [6]. Leaf senescence usually precedes plant death in monocarpic species and facilitates nutrient mobilization to the seeds, i.e., to the next generation. In perennial plants, leaf senescence facilitates whole plant lifespan through nutrient recycling, control of water status, and adaptation to the environment and seasonal changes. In both cases, the main role of leaf senescence is for nutrient management and export to growing sinks.

*Oryza sativa* (rice) is one of the major staple crops and a key nutritional source for half of the world’s population [7]. Then, increased grain yield in rice is crucial to cope with the rising food demands due to global population growth. Rice is a monocarpic plant in which leaf senescence overlaps the reproductive stage; thus, premature senescence can reduce grain yield under adverse environmental conditions [6]. In cereal crops such as rice, all the nutrients are transported predominantly from leaves to grains [8]. Leaves capture solar energy for photosynthetic carbon (C) assimilation and provide grains with C in the form of sugars; decreased photosynthetic activity during senescence reduces C fixation and sugar export to grains. By contrast, nitrogen (N) can be transported to grains through N remobilization, and increased protein breakdown during senescence promotes N remobilization from leaves to grains [9,10]. Subsequently, leaf senescence influences the C/N balance of resource mobilization and allocation to the grains [11]. Delayed leaf senescence can increase grain yield due to continued photosynthetic activity and C fixation, whereas it decreases grain protein content due to delayed N remobilization from the source leaves [11]. Accelerated leaf senescence can increase grain protein content [12]. Thus, the timing of leaf senescence is crucial for regulating grain yield and quality during the grain-filling period in rice.

As genome-wide alterations in gene expression occur during leaf senescence, genes that are controlling leaf senescence in plants are designated as senescence-associated genes (*SAGs*), and many *SAGs* also have been isolated and characterized in rice [13,14,15,16]. They are involved in the degradation of macromolecules, nutrient relocation, defense mechanisms, transcriptional regulation, and signal transduction [1,16]. This review summarizes the recent research developments in rice leaf senescence. It includes knowledge about the chlorophyll degradation pathway, description of stay-green traits, transcriptional regulations, impacts of leaf senescence on N metabolism, the relationship between senescence and stress, and finally, it proposes perspectives for the future.

## 2. Chlorophyll Degradation Pathways in Rice

**Figure 1:**
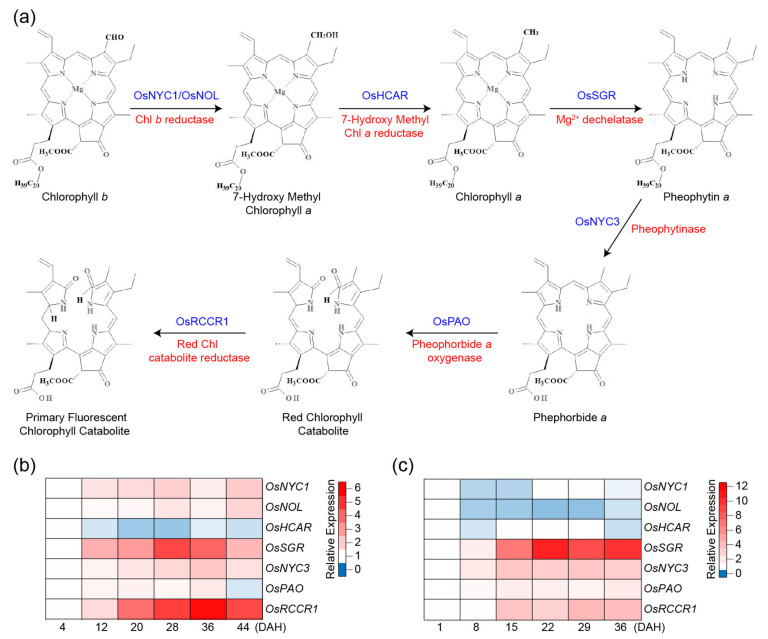
The PAO/phyllobilin pathway of chlorophyll (Chl) breakdown in leaves and in silico analysis of the expression of Chl catabolic enzymes (*CCEs*) in rice flag leaves during the grain-filling stage. (**a**) Enzymatic reactions of the first part of the Chl degradation pathway, from Chl *b* to primary fluorescent Chl catabolite (*p*FCC), that take place inside the chloroplast. See the text for abbreviations of enzyme names. Expression data were obtained from RNA-seq analysis (**b**) [20] and public microarray data (**c**) [21]. DAH; days after heading. Each expression data is normalized to starting time points ((**b**), 4 DAH; (**c**), 1 DAH, respectively). Based on the transcript levels of starting time points, the relative gene expression levels of other time points were quantified. The blue or red colors indicate the lower or higher transcript abundances than those of relevant control (white), respectively.

Leaf senescence is monitored by the gradual loss of green pigments, which is mainly due to chlorophyll (Chl) degradation [4]. Most Chl are present in protein complexes in leaves because it is critical to prevent cells from the photooxidative damages that may be caused by the free Chl molecules if disassociated from the light-harvesting Chl-binding complex proteins (LHCs) of the thylakoids [17]. As Chl degradation is preceded by the degradation of LHCs in senescent leaves [18], the biochemical pathway of Chl breakdown is tightly controlled, and the released Chl is converted to non-phototoxic colorless catabolites (phyllobilins) via the PAO (Pheophorbide *a* Oxygenase)/phyllobilin pathway, and stored in the vacuole (Figure 1a) [19]. This catabolic pathway starts in senescing chloroplasts by the conversion of Chl *b* to Chl *a*, which is a degradable form and is then followed by the removal of the Mg^2+^ ion and the phytol moiety. Subsequently, the cyclic tetrapyrrole is linearized and catabolized to a primary fluorescent Chl catabolite (*p*FCC), which is colorless (Figure 1a). All of these reactions are common in higher plant species and rice, and all Chl catabolic enzymes (CCEs) have been well characterized [17].

The conversion of Chl *b* to Chl *a* requires the action of two enzymes, Chl *b* Reductase (CBR) and 7-hydroxymethyl Chl *a* Reductase (HCAR, Figure 1a). Rice has two CBR, named NONYELLOW COLORING1 (OsNYC1) and NYC1-LIKE (OsNOL) [22,23]. The co-localization of OsNOL and OsNYC1 in the thylakoid membrane, as a heterodimer is required for Chl *b* reductase activity for the conversion of Chl *b* to 7-hydroxymethyl Chl *a* (HMChl *a*) and degradation of light-harvesting complex II during senescence [23]. The second step of the reduction of Chl *b* is the conversion of HMChl *a* to Chl *a*, which is catalyzed by HMChl *a* reductase (HCAR) [17]. The rice genome contains a single HCAR homolog (OsHCAR), and its knockout mutant displays persistent green leaves during senescence, accumulating HMChl *a* and pheophytin *a* (Pheo *a*), which is a downstream intermediate of the Chl breakdown pathway [24]. These results indicate that HCAR may play a pivotal role in protecting plants by inhibiting the accumulation of HMChl *a* and Pheo *a* in leaves [24].

Mendel’s green cotyledon gene, the *Stay-Green* (*SGR*) gene, encodes Mg^2+^-dechelatase, which is responsible for the removal of Mg^2+^ from Chl *a* to yield Pheo *a* [25]. SGR functions as a key positive regulator of Chl degradation by physically interacting with the CCEs and LHCII proteins [26,27]. The rice *ossgr* mutants typically show a strong stay-green phenotype during both natural and dark-induced senescence because of the stable Chl-protein complex and thylakoid membrane structures [28,29]. Despite their greener phenotypes, the *ossgr* mutants show the same decrease in photosynthetic capability during senescence as wild type (WT), leading to no yield advantage [28,29,30]. An SGR rice homolog, SGR-like (SGRL), also displays Mg^2+^-dechelatase activity, but against the chlorophyllide *a* precursor and not against Chl *a* [25]. The *OsSGRL* gene is downregulated during senescence, and its overexpression promotes Chl degradation and reduces the Chl-binding protein levels [31].

The removal of the phytol chain of Pheo *a* increases the solubility of the further breakdown products and is catalyzed by the Pheophytinase (PPH), which yields the highly phototoxic intermediate pheophorbide *a* (Pheide *a*) [32]. The Non-Yellow Coloring 3 (OsNYC3) pheophytinase is responsible for this step in rice [33]. The PAO converts the toxic Pheide *a* to a red Chl catabolite (RCC), and further to the primary fluorescent Chl catabolite (*p*FCC) that is generated by the Red Chl Catabolite Reductase (RCCR) [34]. Since the colored intermediates Pheide *a* and RCC are phototoxic, the knockdown and knockout mutations in *OsPAO* and *OsRCCR1* generate cell death symptoms [35,36]. The *p*FCC catabolites are exported from the chloroplast, modified in the cytosol/ER, and then transported into the vacuole where they accumulate as non-fluorescent Chl catabolites [37].

Analyses of *CCE* expression patterns can infer their functions during senescence. Their transcript levels in rice were analyzed using publically available RNA-seq data [20] and microarray data [21] derived from flag leaves grown under natural field conditions (Figure 1b, c). The results show that the expressions of *OsNYC1*, *OsNOL*, *OsSGR*, *OsNYC3*, *OsPAO,* and *OsRCCR1* were higher during the grain-filling stage, but *OsHCAR* transcripts were more abundant in the early grain-filling stage. Most of the CCEs rice mutants-display-stay-green phenotypes during natural- and/or artificially induced-senescence that are discussed in the next section.

## 3. Stay-Green Traits in Rice

Leaf senescence is easily observed because Chl degradation leads to leaf yellowing [38]. Stay-green is the heritable delayed senescence phenotype observed in comparison with a reference genotype, and *stay-green* mutants refer to genotypes that retain green leaves during senescence [39,40]. Maintaining leaf Chl levels and active photosynthetic capacity extends the time for assimilation processes and grain development, thereby increasing crop yield [41,42]. The genetic and physiological ways to obtain seemingly similar stay-green phenotypes are diverse, and five types of stay-green plants have been reported [39]. Their differences are based on the Chl behavior during senescence and their photosynthetic activities [39,43].

The Type A stay-green plants initiate senescence lately but lose Chl at a normal rate. In Type B stay-green plants, senescence is started on schedule, but thereafter, the decline of the photosynthetic activity is comparatively slower than in WT. In Type C stay-green plants, Chl may be retained more or less indefinitely because of defects in pigment breakdown, but functional senescence occurs on a normal time-scale beneath the cosmetic surface of retained pigmentation. Therefore, Type C stay-greens are named cosmetic stay-green. The Type D stay-greens are described as pseudo-stay-greens because their leaves die before or in the middle of the senescence process as if freezing, and subsequently, the plant still appears green after death. The Type E stay-green plants accumulate higher levels of Chl in photosynthetic tissues, resulting in a delay of senescence and the conservation of green tissue. However, the photosynthetic capacity of Type E stay-greens follows the normal ontogenetic pattern. Both Type A and B stay-green plants are termed functional stay-greens as the duration of the photosynthetic capacity is extended. These stay-green types are sought by breeders as they can result in higher yield. Several studies have reported a synergistic effect by combining functional stay-green and other useful traits such as tolerance to drought and heat stress [44]. On the contrary, the Type C, D, and E plants are categorized as non-functional stay-green that retain greenness due to the impairment of Chl catabolism but lack the maintenance of leaf functionality during senescence [39,45]. Most CCE mutants in rice display a stay-green phenotype during senescence but lack an extended photosynthetic capacity and grain yield advantage; therefore, these mutants are regarded as non-functional stay-green plants.

Due to the high importance of maintaining photosynthesis longer and/or in promoting nutrient recycling and mobilization throughout the plant, the stay-green traits were investigated in many plant species, including rice, maize, Arabidopsis, soybean, barley, tomato, pepper, and wheat, and they were used as markers in plant breeding programs [46,47,48,49,50,51,52].

Several studies report that the Chl content in rice is regulated by a quantitative trait locus (QTL) [47,53,54,55]. Analyses of Chl content and the degree of greenness in the flag and second leaves identified 46 QTLs that were associated with delayed senescence [53]. Cha et al. mapped the *stay-green* (*sgr*) locus, which is responsible for maintaining greenness during leaf senescence, but not photosynthetic activity, to the long arm of chromosome 9 [47]. Further studies identified *SGR* as a senescence-associated gene encoding a novel chloroplast protein and Mg^2+^-dechetalase mediating Chl breakdown, which confirmed that *sgr* was a cosmetic stay-green locus [28,30].

A total of six QTLs (csfl2/tcs2, tcs4, tcs5, csfl6, csfl9/tcs9, and csfl12) were identified in recombinant inbred line populations derived from intra- and inter-subspecific crosses of rice varieties [56]. The functional stay-green *japonica* rice ‘SNU-SG1′ is mapped on chromosome 9. The presence of SNU-SG1 improved grain yield by maintaining photosynthetic activity in the flag and second leaves during grain filling, and by increasing sink strength due to high seed-setting rate [54]. Fine mapping performed by Lim et al. identified eleven main-effect loci in the *japonica* rice SNU-SG1 that were responsible for functional SG phenotypes [57]. Fifteen strong candidate genes were identified that explained these main-effect QTLs using the whole-genome sequence [57]. All these genes represent interesting candidates for further plant breeding. The *qCC2* major QTL for Chl content was identified using a population derived from an interspecific cross between *O. sativa* and *O. grandiglumi* [55]. The *GW2* gene that encodes an E3 ubiquitin ligase is located in the *qCC2* region and is responsible for leaf senescence through the transcriptional regulation of phytohormone signaling pathways [55]. Whether the *qCC2* QTL is explained by *GW2* allelic variations in *O. sativa* and *O. grandiglumi* remains to be determined. Ten stable QTL regions for six delayed senescence traits have been identified [58]. Expression analysis of five candidate genes displayed the differential transcript levels, suggesting their strong association with the senescence pattern in the flag and second leaves and possible contribution in enhancing grain yield through genomics-assisted breeding.

A genome-wide association study (GWAS) of a diverse worldwide collection of rice accessions identified forty-six significant association loci in natural variation of Chl content (CC) [59]. Among them, the *Grain number, plant height, and heading date7* (*Ghd7*) was a major locus for natural variations of Chl content that was involved in the repression of Chl and chloroplast biosynthetic genes [59]. As indicated by its name, this locus is a functional stay-green locus that controls also grain number, plant height, and heading date. Another candidate revealed by GWAS is *Narrow leaf1* (*NAL1*) [59]. A high degree of polymorphism in the 5′ UTR and four non-synonymous single nucleotide polymorphisms (SNPs) in the coding region of *NAL1* was shown to confer natural variations of Chl content [59]. Whether pleiotropic effects of *NAL1* in regulating flag leaf width and spikelet number per panicle might be helpful for rice breeding remains to be determined. In another GWAS study, 25 known genes for CC and stay-green (SG) traits were identified in natural rice variations [60]. Non-synonymous SNPs were detected in six of the genes and three SNPs in the promoter region of *OsSG1* [60]. Another GWAS revealed fourteen regions associated with CC and strong SG phenotypes [60]. From them, twenty-five candidate genes identified around the GWAS signals are genes with known important roles in CC and SG phenotypes. This suggests that these genes could be involved in the genetic variation of CC or SG. Non-synonymous SNPs within six of these genes, and three SNPs in the promoter of *OsSG1,* were unveiled. The impact of each of these genes on plant performance remains to be explored.

Two representative rice subspecies, *japonica* and *indica*, display distinct morphological and physiological features as well as clear variation in genomic sequences [61]. Furthermore, *indica* displays early leaf senescence, whereas *japonica* displays late leaf senescence [62]. Then, QTL mapping was conducted to determine the genetic factors responsible for the differential senescence patterns between *indica* and *japonica* [30]. Results showed that allelic polymorphisms in the *OsSGR* promoter in *indica* result in higher and earlier induction of *OsSGR*, thereby triggering earlier senescence. The introgression of *japonica OsSGR* alleles into elite *indica* cultivars produced near isogenic lines (NILs) with delayed leaf senescence and extended photosynthetic competence, leading to improved grain filling rate and yield. Then, the *japonica OsSGR* alleles represent technical solutions for a beneficial breeding strategy in rice.

In addition to QTL and GWAS approaches, experiments leading to the characterization of mutants were carried out. The screen of ethyl methansulphonate (EMS)-induced rice mutants led to the identification of three stay-green mutants that also displayed drought tolerance [63]. Amongst them, *SGM-3* was characterized as a novel functional stay-green mutant with extended photosynthetic capacity during senescence, increased harvest index, and higher grain yield under irrigated as well as drought conditions which can be further used for the development of high-yielding lines [63]. The exact function of SGM-3 remains to be determined.

In summary, we can see that both the quantitative genetics and mutagenesis approaches successfully identified several functional stay-green genes that play a role in plant productivity. Although the function of many of these genes and loci still remains to be elucidated, their positive alleles could be used in marker-assisted plant breeding programs.

## 4. Transcriptional Regulation of Leaf Senescence

Leaf senescence is an age-dependent process and occurs irrespective of environmental conditions. Its initiation is tightly regulated by endogenous factors including phytohormones and metabolic status. Nevertheless, exogenous factors such as light, drought, nutrient deficiency, and pathogen can modulate the onset and the intensity of the leaf senescence symptoms [64]. Nutrient and water deficiencies, as well as shading, are known to accelerate leaf senescence as the absence of nutrients promotes proteolysis and the recycling of plant resources. As such, dark stress and nitrogen-starvation have been used as senescence-inducing factors to screen leaf senescence mutants in Arabidopsis.

One informative way to understand leaf senescence and the different factors that control it is to identify the senescence-related transcription factors (TFs), which can act as nodes in gene expression pathways of the senescence-associated genes (*SAGs*) and modulate *SAG* expressions directly or indirectly by binding to the *cis*-elements in their promoter regions [1,65]. Phytohormones are endogenous factors involved in complex interactions that depend on the successive stages of leaf development. Ethylene, abscisic acid (ABA), jasmonic acid (JA), auxin, and salicylic acid (SA) promote senescence, while cytokinin and gibberellin delay senescence [66]. Thus, it is crucial to identify TFs acting downstream of the phytohormonal signaling networks and the interactions between TFs and phytohormones to understand the molecular mechanisms regulating senescence. Several of the TFs cited in this review and listed in Table 1 indeed integrate phytohormonal signals to modulate leaf senescence [67].

The *NAC* (NAM, ATAF1/2 and CUC2) gene family is one of the largest group of plant-specific TFs, with more than 150 genes in rice, and a large set of *NAC* factors are involved in leaf senescence [68]. These NAC proteins contain a conserved NAC domain at the N-terminal region for DNA-binding at the N-terminal region and variable transcription regulatory regions (TRRs) at the C-terminal region acting as a transcription activator or repressor [69]. In Arabidopsis, 57.5% of the *NAC* genes are differentially expressed during leaf senescence, and they positively or negatively regulate leaf senescence [70]. In other than Arabidopsis, including rice, the senescence-associated *NACs* remain largely unknown. However, the few examples cited below show that several of the rice *OsNAC* factors control leaf senescence and respond or participate in hormone signaling, and especially in response to ABA. As such, the *OsNAP* gene, which is the homologous gene of the Arabidopsis *AtNAP*, is highly expressed in the senescing tissues and directly regulates *SAGs* in response to ABA [71,72,73]. The *OsNAP*-overexpressing transgenic rice show an early leaf senescence phenotype, whereas the RNA-interference (RNAi) lines display delayed leaf senescence with an increase in grain yield, indicating that OsNAP functions as a senescence-promoting TF [71].

ONAC2, which is homologous of AtORE1, promotes leaf senescence by inducing ABA biosynthetic genes, downregulating ABA catabolic genes, and regulating the Chl degradation genes [74]. Therefore, the overexpression of *ONAC2* causes early leaf senescence, while *ONAC2* RNAi lines exhibit delayed senescence phenotype along with a 10% increase in seed productivity. ONAC096 positively regulates Chl degradation and the expression of several *SAGs* during leaf senescence and mediates ABA-induced leaf senescence [65]. ONAC54 is also tightly associated with the regulation of ABA-induced leaf senescence; *ONAC054*-overexpressing plants display early leaf yellowing, whereas *onac054* knockout mutants maintain green leaves longer [64]. Intriguingly, ONAC054 nuclear import is regulated by the cleavage of a C-terminal putative transmembrane domain through alternative 3′ splicing, showing that ONAC054 is important for ABA-induced leaf senescence and is itself controlled by multilayered regulatory processes. OsNAC109 regulates the transcription of senescence- and hormone-associated genes by binding to NAC recognition sequence element (CNTCSSNNSCAVG) within promoter regions [75]. The knockout mutants of *ONAC109* show premature senescence with altered expressions of senescence and photosynthesis-related genes. The ONAC106 that negatively regulates natural senescence, by directly modulating the expression of several *SAGs* such as *OsSGR, OsNYC1,* and also *OsNAC5*, was shown to respond not to ABA but to salt stress [76]. The overexpression of *ONAC106* delays leaf senescence under natural and dark-induced conditions, and its role in response to salt stress remains to be investigated. The ONAC011 is one of the positive regulators of leaf senescence. Although the ONAC011 regulatory pathway, including signals and target genes, remains unknown, it was shown that ONAC011 accelerates heading time during the reproductive phase and then possibly modulates plant productivity and acts as a positive regulator of leaf senescence in rice [77]

The transcriptome analysis of field-grown flag leaves during senescence has revealed six other NAC TFs that are upregulated from vegetative to senescence stages (*OsNAC1*, LOC_Os02g36880; *ONAC39*, LOC_Os03g21030; *OsNAP*, LOC_Os03g21060; *ONAC010*, LOC_Os07g37920; *OsNAC18*, LOC_Os07g48450; *ONAC121*, LOC_Os10g421300) [78]. However, except for *OsNAP*, their functional roles in leaf senescence are still unknown [78].

The MYB TFs form the largest TF family in rice (at least 197 members) and function as key regulators of plant development in response to biotic and abiotic stress [79]. Similar to the senescence-associated NAC TFs, the MYB TFs are not only influenced by age but also by hormones and for some by abiotic stresses. The MYB-related rice TF RADIALIS-LIKE3 (OsRL3) promotes dark-induced leaf senescence and delays the response of rice plants to salt stress via the ABA signaling pathways [80]. Under dark-induced senescence conditions, the *osrl3* mutants exhibit a stay-green phenotype due to the reduced expressions of Chl degradation and *SAGs*. OsMYB102 functions as a negative regulator of leaf senescence [81]. The overexpression of *OsMYB102* delays senescence under natural, dark, and ABA conditions, while the *osmyb102* knockout mutant shows accelerated senescence [81]. The transcript levels of several *SAGs* and ABA-related genes are altered in the *OsMYB102*-activation tagging lines, indicating that OsMYB102 plays a critical role in leaf senescence by downregulating ABA accumulation and ABA signaling responses.

The plant-specific WRKY TFs are involved in many biological processes, and they particularly respond to and mediate stress responses [82]. According to microarray data from rice flag leaves, we can see that among the 89 *WRKY* members identified so far, several are induced during leaf senescence [82,83]. The relationship to senescence was investigated in depth for a few of them. For example, *OsWRKY80* is upregulated under dark-induced senescence and drought stress, as well as by ABA treatment [84]. The overexpression of *OsWRKY23* in Arabidopsis was shown to enhance dark-induced leaf senescence [85]. OsWRKY42 represses OsMT1d-mediated scavenging of reactive oxygen species (ROS), thereby promoting leaf senescence [86]. *OsWRKY5* is upregulated at the onset of leaf senescence, and its overexpressing line displays early leaf yellowing under aging and dark treatment, while the *oswrky5*-knockdown mutants show the opposite phenotype [87]. OsWRKY5 acts as a positive regulator of leaf senescence in rice and regulates the expression of Chl degradation genes, *SAGs*, senescence-associated *NAC* genes (*OsNAP* and *OsNAC2*), and ABA biosynthetic genes.

Shading or dark treatment are known to enhance leaf senescence. The plant-specific phytochrome-interacting factors (PIFs) are basic helix–loop–helix (bHLH)-type TFs that regulate various biologic processes in a red-light and phytochrome (phy)-dependent manner, including leaf senescence and Chl biosynthesis [88,89]. OsPIL1 is promoting Chl biosynthesis via trifurcate feed-forward regulatory loops that involve two GOLDEN2-LIKE (OsGLK) TFs [90]. The *ospil1* mutants exhibit earlier senescence during dark treatment, indicating that OsPIL1 negatively regulates leaf senescence in rice [90]. Microarray analyses unveiled that several *SAGs* were upregulated in *ospil1* mutants, while the *OsGLKs* negative regulators of leaf senescence were strongly repressed [91].

OsTZF1 (also known as OsDOS; Delay of the Onset of Senescence) is a CCCH-tandem zinc finger protein that acts as a negative regulator of leaf senescence under various stress conditions as salt stress, drought, and dark [92]. The overexpression of *OsTZF1/OsDOS* delays leaf senescence, whereas RNAi knockdown causes accelerated age-dependent leaf senescence [93]. OsTZF1 confers abiotic stress tolerance through the downregulation of several stress-related genes [92] and controls leaf senescence in a JA-dependent manner [93].

In addition to the identification of the different TFs involved in leaf senescence, one of the main challenges is to understand their interconnections and networks. If network analyses are well advanced in Arabidopsis, we still are at the beginnings in rice. However, a few studies performed in rice reveal such interconnections. As such, the ETHYLENE RESPONSE FACTOR 101 (OsERF101) that positively regulates leaf senescence was shown to enhance the expression of *OsNAP* and the JA-responsive *OsMYC2* TF [94]. The overexpression of *OsMYC2* promoted leaf senescence and reduced Chl content under darkness, indicating that OsMYC2 functions as a positive regulator of leaf senescence [95]. The *oserf101* knockout mutant accordingly exhibits delayed leaf senescence with higher Chl content during dark-induced and natural senescence [92]. After JA treatment, the leaves of *oserf101* still contain more Chl than WT, indicating that OsERF101 is involved in promoting JA-induced leaf senescence. The expression of the JA signaling genes is indeed downregulated in *oserf101.*

Although not exhaustive, the several examples presented above show that the different TFs identified so far in rice can regulate positively or negatively the onset of leaf senescence, in response to one or several endogenous and/or exogenous signals. Many of these TFs are implicated in the response to ABA, JA, ethylene, SA, and dark. While their interconnections and networks remain poorly known and deserve further studies, it is already noticeable that several of the TFs listed in Table 1 have some senescence-related TFs as direct downstream target genes. Strikingly, we can see in Table 1 that *OsNAP* is a downstream target of many of them [71,72,73]. Dissecting the leaf senescence regulatory network will permit the identification of the master regulators and then facilitate the choice of candidate genes for further investigations. Nevertheless, a better characterization of the phenotypes of the senescence-TFs overexpressors and knock-down lines, based on the evaluation of plant performances, flowering dates, heading time, yield, and stress resistance, should point to the best candidates for plant breeding strategies.

## 5. Leaf Senescence and Nitrogen Metabolism

Nitrogen (N) is essential for all living organisms and is predominantly remobilized from source to sink organs during leaf senescence [96]. During the vegetative growth stage, chloroplasts contain up to 75–80% of the leaf N, primarily as photosynthetic proteins including thylakoid proteins, light harvesting complexes, photosystems, Calvin cycle proteins, and Rubisco [2,97]. In rice, 70–90% of total panicle N is originating from the remobilization of N from vegetative organs [9]. Chl degradation during leaf senescence enables or signals further catabolic processes in chloroplasts [18]. The subsequent dismantling of chloroplasts is a major N source for recycling and remobilization [18]. Rubisco and other photosynthetic proteins are degraded during senescence, and the released N is remobilized step by step from source leaves to sink leaves, and then ultimately to grains [97]. The remobilization of N from aging tissues to the seeds is an important determinant of productivity and yield, especially under N limitation [98].

Autophagy (meaning self-eating) facilitates the degradation of unwanted cytoplasmic components inside the lytic vacuoles [99]. Two types of autophagy have been described in plant leaves so far. The macro-autophagy is a vesicular process that involves eighteen ATG proteins in the formation of a double membrane vacuole named the autophagosome. Autophagosomes are formed in the cytosol where they engulf unwanted cytoplasmic materials (called cargoes) and drive them to the lytic vacuole. By fusing their external membranes to the tonoplast, autophagosomes release their inner membranes sequestering cargoes inside the vacuole for degradation [99]. Micro-autophagy consists of the direct engulfment of cytoplasmic material inside the vacuole through tonoplast invagination. Macro-autophagy is involved in the degradation of the stroma proteins released from the chloroplast stromules in budding structures named Rubisco-containing bodies (RBC). The trafficking of RBC to the lytic vacuole is dependent on the macro-autophagy machinery [100]. Entire damaged chloroplasts can also be degraded in the vacuole through a micro-autophagy process [101]. In Arabidopsis, it was shown that macro-autophagy is essential for the remobilization of several nutrients such as N, sulfur, and iron [102,103,104]. The ATG8 protein is essential for autophagosome membrane formation and for cargo sequestration. The ATG8 proteins interact with the cargoes directly, or indirectly through receptors, and facilitate their sequestration in the autophagosomes. The constitutive overexpressing of *ATG8* isoforms in Arabidopsis has been shown to stimulate the autophagic activity and to increase N remobilization from the rosette leaves to the seeds [105].

In rice, thirty-three Autophagy-related genes (*OsATGs*) have been identified [106]. OsATG7 is essential for autophagosome formation and participates in efficient N utilization and remobilization [97]. The *osatg7-1* mutant display poor biomass and NUE during vegetative growth. Unable to export N from senescing leaves, this mutant is sterile. As the transcript levels of *OsATG8a*, *OsATG8b*, and *OsATG8c* are increased under N starvation, their overexpression was assayed in rice [107,108,109,110]. Irrespective of the OsATG8 isoforms, rice transformants display higher NUE and yield [107,108,109,110]. The *OsATG8a*-overexpressing lines accumulate more N in their grains and less in their dry remains compared to WT, which indicates a better N remobilization to the seeds that increase grain yield [107]. The overexpression of *OsATG8b* could improve NUE and increased grain yield by stimulating autophagy flux and by enhancing the activities of enzymes related to N metabolism [110]. The higher N remobilization efficiency of *OsATG8b*-overexpressors was demonstrated using ^15^N labeling and tracing assays. On the contrary, the *osatg8b* knockout mutants showed the opposite phenotypes [110]. The specific roles of the different ATG8 isoforms in Arabidopsis and rice remain to be explored, as well as their respective involvement in nutrient recycling. The contribution and the nature of the plant proteases involved in the last steps of the autophagy process are still largely unknown [111]. Nevertheless, autophagy and senescence-related proteases are essential for the remobilization of N, as they facilitate the release of a pool of free amino acids from the protein cargoes that can be exported through the phloem to the sinks and up to the seeds.

Glutamine (Gln) and asparagine (Asn), which contain two N atoms per molecule, are considered the main amino acids involved in N translocation in the phloem sap [112]. From the bulk of amino acids originating from autophagic protein degradation, it is likely that amino-acid interconversions and transaminations occur to facilitate phloem loading [112]. Especially, the synthesis of Gln and Asn in senescing organs appears essential for N remobilization [113,114]. The increase in the Gln/glutamate (Glu) and Asn/aspartate (Asp) ratios with leaf senescence is in line with this assumption [2]. Therefore, attention has been paid for a long time to the role of the glutamine synthetases (GS) and asparagine synthetases (ASN) for the re-assimilation of ammonium in old leaves [115]. The lack of OsGS1;1, which is the major cytosolic isoform of GS in rice, caused a shortage of N resources in senescing source organs and limited N remobilization into sink organs, resulting in severe growth retardation and lower grain yield [115]. Glutamate synthetase (GOGAT, also known as glutamine-2-oxoglutarate aminotransferase) catalyzes the transfer of the amide group of Gln (formed by GS) to 2-oxoglutarate (2-OG) to synthesize two molecules of Glu [116]. The GS/GOGAT cycle is the major route for ammonium assimilation and sustains N metabolism in higher plants [116]. It was recently shown that when the expression of NADH-dependent *OsGOGAT1* is enhanced, the remobilization of N from young leaves to old leaves is increased, which causes early leaf senescence but increases N remobilization, leading to higher N content in grains [96]. Interestingly, the mutation in the Ferredoxin-dependent OsFd-GOGAT also leads to premature leaf senescence and facilitates N remobilization [117,118]. This shows that the NADH- and Fd-dependent GOGATs play opposite roles in rice, the former being involved in N recycling and remobilization, the latter being involved in N primary assimilation and photorespiration. Asn synthesis is catalyzed by Asparagine synthetase (ASN) through the transfer of the Glu-amide group to the amide position of Asp. Asn has an essential role for N storage and transport in plants [119]. Among the two rice *ASN* genes, OsASN1 is essential for plant growth under both N-sufficient and N-limiting conditions, and for biomass and grain yield [120]. During the grain-filling stage, the *osasn1* mutants accumulate more N in their flag leaves and less N in panicles than the wild type, indicating reduced N remobilization from source to sink organs.

**Figure 2:**
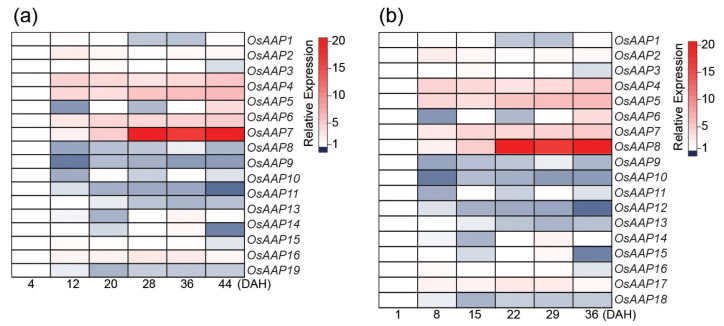
Heat map of the expression of the rice *AAPs (amino acid permease, OsAAPs*) during leaf senescence. Expression data were from RNA-seq analysis (**a**) [20] and microarray data (**b**) [21]. Gene nomenclature of each gene was followed in the previous report [120]. Relative expression levels were quantified as describes in Figure 1.

One important step for N-remobilization during leaf senescence is phloem loading and amino acid transport [112]. Many senescence-associated amino acid transporters have been identified in Arabidopsis and rice, but their specific roles remain largely unknown, even in Arabidopsis [11,121]. It was shown in Arabidopsis that the AAP8 (Amino Acid Permease 8) plays a key role in amino acid phloem loading and its function strongly affects sink size and number [122]. Interestingly, a recent paper shows that increasing the expression of the rice *OsAAP3* that facilitates the transport of arginine and lysine into the mesophyll cells influences leaf senescence [123]. The overexpression of *OsAAP3* decreases grain yield and leads to lesion mimic and leaf senescence in rice flag leaves by an increase of nitric oxide. In a previous study, the authors have shown that blocking OsAAP3 promotes rice tillering, grain yield, and tiller bud elongation [124]. Together, these studies show that enhancing or inhibiting amino acid influxes or effluxes in leaves impacts senescence program, leaf longevity, and plant productivity. The rice genome harbors nineteen *AAP* genes [125], and previous reports had shown the amino acid transporter activities of OsAAP1, OsAAP4, OsAAP5, and OsAAP6, which play important roles for grain quality and yield [126,127,128,129]. Using publically available expression data, we analyzed the transcript levels of the rice *AAP* genes (Figure 2). The expressions of *OsAAP4*, OsAAP5, *OsAAP6*, *OsAAP7*, and *OsAAP8* are upregulated during the grain-filling stage in rice, suggesting their possible role for N-remobilization during senescence. Then, their function during leaf senescence may deserve attention.

## 6. Stress-Induced Senescence

As plants are sessile, they are frequently exposed to adverse environmental conditions and are unavoidably challenged by various biotic and abiotic stresses during the growing season. Abiotic stresses such as drought, floods, salinity, radiation, darkness/shading, and temperature changes, nutrient deficiency, and mineral toxicity adversely affect plant growth and thereby cause the loss of productivity [5]. Attacks by diverse pathogens such as fungi, bacteria, nematodes, oomycetes, and insects are included in biotic stress [130]. Therefore, narrowing the yield gap between optimal growth and adverse environmental conditions throughout the globe is an urgent challenge to ensure food security and safety in the coming years [131].

Under harsh and unfavorable conditions, plants are acclimated using various ways, including conferring tolerance to environmental stresses or accelerating the senescence (stress-induced senescence) to ensure their survival [132]. Although levels of gene regulation and transcriptome changes by developmental and stress-induced senescence are not entirely overlapping, age-dependent and stress-induced senescence share many signaling pathways via TFs, the involvement of phytohormones such as ABA, JA, and SA, which also synthesize during stress conditions, and physiological, biochemical, and molecular mechanisms [5,94,132]. For example, the expression of *OsNAP* was significantly induced by ABA and abiotic stress, and hence, the overexpression of *OsNAP* results in improved tolerance to abiotic stresses such as high salinity, drought, and low temperature, indicating that OsNAP function as a transcriptional activator in mediating abiotic stress responses as well as leaf senescence [71,73]. OsNAC2, which functions as a positive regulator of senescence, directly activates the expression of Chl degradation genes and *SAGs,* and hence, its overexpression causes accelerated leaf senescence [74]. The knockdown of *OsNAC2* leads to increased tolerance to drought and salinity stresses by the upregulation of stress-related and abscisic acid (ABA)-signaling genes [133]. The expression of *OsNAC066* is significantly enhanced by multiple abiotic factors, and its overexpressing transgenic lines improve tolerance to drought and oxidative stress, indicating a positive regulator of them [134]. However, its role for senescence remains still unknown. It has been shown that OsWRKY93 was involved in both flag leaf senescence and response to biotic stress [135]. *OsWRKY93*-overexpressing plants show increased resistance to blast disease by the enhancement of ROS production and PAMP-triggered immune response, indicating that OsWRKY93 can be a favorable candidate for the breeding of high-yield and disease-resistant rice [135].

Global climate change that might affect crop production drastically is regarded as one of the future challenges, and it affects agriculture in different abiotic and biotic ways such as increasing the average temperature, heat stress, variations in annual rainfall, pests, or microbes, global change of atmospheric CO_2_ or ozone level, and fluctuations in sea level [136]. Therefore, the development of climate-resilient crop species that will have enhanced and sustained productivity traits is becoming important for ensuring global food security [137]. For instance, high temperature during the grain-filling stage can cause a nearly 50% reduction in rice yield [138], and heat-tolerance studies in rice have mainly focused on the reproductive stage due to its high sensitivity and critical influence on grain yield [139]. Plants respond to high temperature through the activation of the heat shock TFs (HSFs) or other stress-related genes [140,141]. Heat stress-responsive two NAC TFs, *ONAC127,* and *ONAC129* are specifically expressed in the pericarp of rice seeds [141]. They are involved in the apoplasmic transport of photosynthates for starch accumulation during grain filling as a heterodimer.

Due to climate warming, plants are allowed to grow for a longer time during each growing season. Rice especially in Southeast Asia may increase the growth potential grown in the open paddy fields by absorbing more CO_2_ for photosynthesis and hence increase the grain yield, but plant growth is also restricted by shorter day length in autumn, regardless of warming [142,143]. Thus, it is important to analyze how to adapt the plant according to the variable growing season length by adjusting the length of vegetative and reproductive growth, senescence duration, and timing of heading [5]. For example, adoption of the functional stay-green trait may be one of the ways to deal with future warming conditions by extending the time for photosynthesis and further minimizing the sink limitation, leading to higher yield.

## 7. Breeding Strategies to Improve Rice Yield

The previous sections present different approaches to identify senescence genes that could be candidates for plant breeding strategies. Amongst these genes, we can distinguish the TFs that regulate positively or negatively the onset of the leaf senescence, and the executioners that work to the preservation of cell longevity or in the successive degradation events that allow the recycling of nutrients. Modulating both kinds of genes could influence leaf longevity and therefore impact plant productivity.

The first attempt to identify regulators or executioners in Arabidopsis has been done by analyzing the transcriptomic data of senescing leaves [144,145]. This facilitated the identification of senescence repressed or senescence-associated genes that were either regulators or executioners. In rice, senescence-associated genes were also identified [13,16], and studies of their roles were performed using reverse genetics. Studies of mutants or overexpressors mainly focused on executioners or TFs that had also been identified in other plant species; for example, the senescence-associated N enzymes and the autophagy proteins are known to participate in N remobilization, and the homologous of *OsNAP* and *OsNAC2* control leaf senescence.

The identification of new regulators/actors involved in leaf senescence was carried out using quantitative genetics (either QTL or GWAS). Quantitative traits considered were chlorophyll content, leaf senescence onset, and agronomic performances. Although such a forward genetic approach provided many cosmetic stay green candidates, it also revealed functional stay green candidates that might serve in traditional marker-assisted breeding programs, such as for example in the case of the introgression of the *japonica OsSGR* alleles into the elite *indica* cultivars that resulted in a higher grain-filling rate and better yield [30]. Nevertheless, the characterization of many of the QTLs and underlying genes still necessitates efforts. Indeed, the use of GWAS and QTL results for plant engineering requires that the genes underlying the QTLs are identified and confirmed. In addition, as leaf senescence and plant yield are multigenic traits, gene stacking might be considered.

## 8. Conclusions

Senescence and stay-green phenotypes are important traits in rice breeding programs. During the last few years, there have been significant advances in understanding the mechanisms and processes underlying rice leaf senescence. These include the dissection of the Chl degradation pathways, the characterization of stay-green traits, the investigation of the transcriptional regulations and interaction between TFs and phytohormones, and the dissection of impacts on nutrient remobilization and rice yield production. In rice, as in many other monocarpic cereals, grain production is concomitant with plant senescence, and the seed maturation ends with plant death. Therefore, the timing of the senescence onset, the duration of the senescence period, and the intensity of the senescence-related cell degradation are important traits that control the number of offspring in rice, and then grain quality and quantity. Up to now, studies have mainly considered leaf senescence onset. New studies on the duration of leaf senescence may identify additional important genes involved in leaf senescence and plant productivity. Dissecting the genetic bases of the different steps of leaf senescence in rice is of great importance, as it can provide genetic technical solutions for the improvement of rice performances.

The developments of high-throughput transcriptome analyses on mutants and overexpressors of senescence-related TFs should generate insights into the functional and regulatory aspects of leaf senescence in rice. The proteomic and metabolic approaches, which are still less developed, should, in the combination of multi-omics analyses, help to unveil better characterization of the different steps of the leaf senescence program. Functional stay-green traits should be dissected in depth to unveil the mechanisms that cause the extension of photosynthetic capacity. Co-operation of the recent development of non-invasive phenotyping approaches and CRISPR/Cas9-based genome editing can be used to manipulate candidate genes and identify the new regulatory genes and design networks more easily. Phenomic approaches will also facilitate the development of forward genetics approaches to dissect leaf senescence regulation and identify novel genes.

## Figures and Tables

**Table 1:** Summary of transcription factors associated with rice leaf senescence.

Gene	Locus Number	Gene Family	Role in Senescence	Pathways Involved	Direct Downstream Targets	References
*OsNAP*	LOC_Os03g21060	NAC TF	Positive	Age, ABA, dark, salt, drought	OsSGR, OsNYC1, OsNYC3, OsRCCR1, Osh36, OsI57, Osh69, OsI85	[71,72,73]
*OsNAC2*	LOC_Os04g38720	NAC TF	Positive	Age, ABA, dark	OsSGR, OsNYC3, OsNCED3, OsZEP1	[74]
*ONAC106*	LOC_Os08g33670	NAC TF	Negative	Age, dark, salt	OsSGR, OsNYC1, OsNAC5, OsNAP, OsEIN3, OsS3H, OsDREB2A, OsLEA3, OsbZIP23, LPA1	[76]
*ONAC011 (OsY37)*	LOC_Os06g46270	NAC TF	Positive	Age		[77]
*ONAC096*	LOC_Os07g04560	NAC TF	Positive	Age, ABA	OsSGR, OsPAO, OsNYC3, OsRCCR1, OsI85, Osl2, Osl57, OsNAP, ABI5, OsEEL	[65]
*ONAC054*	LOC_Os03g02800	NAC TF	positive	Age, ABA, JA	OsNYC1, OsABI5,	[64]
*OsNAC109 (OsYL3)*	LOC_Os09g38000	NAC TF	Negative	Age, ABA, dark	OsNAP, OsNYC3, OsEATB, OsAMTR1, OsZFP185, OsMPS, OsGA2ox3	[75]
*OsRL3*	LOC_Os02g47744	MYB TF	Positive	ABA, salt, dark	OsSGR, OsNYC1, OsRCCR1, Osl2, Osl43, OsSAG12-2, OsRK1, OsRAB16C, OsRAB16D	[80]
*OsMYB102*	LOC_Os06g43090	MYB TF	Negative	Age, ABA, dark	OsSGR, OsNYC1, OsABF4, OsNAP, OsCYP707A6	[81]
*OsWRKY80*	LOC_Os09g30400	WRKY TF	Positive	Dark, ABA, drought,		[84]
*OsWRKY23*	LOC_Os01g53260	WRKY TF	Positive	Age, dark, SA, pathogen		[85]
*OsWRKY42*	LOC_Os02g26430	WRKY TF	Positive	Age, ROS	OsMT1d	[86]
*OsWRKY5*	LOC_Os05g04640	WRKY TF	Positive	Age, dark, ABA	CCEs, SAGs, OsNAP, OsNAC2, OsNCED3, OsNCED4, OsNCED5	[87]
*OsPIL1*	LOC_Os03g56950	bHLH TF	Negative	Age, dark	OsPORB, OsCAO1, OsGLK1, OsGLK2	[90,91]
*OsMYC2*	LOC_Os10g42430	MYC TF	Positive	JA, dark	Several SAGs.	[95]
*OsERF101*	LOC_Os04g32620	ERF TF	Positive	Age, ABA, drought, JA	OsNAP, OsMYC2, OsJAI1, OsCOI1a, OsSGR, OsNYC1, OsNYC3	[94]
*OsTZF1*	LOC_Os01g09620	CCCH-type zinc finger TF	Negative	Age, ABA, JA, SA, salt, drought, dark	Stress-responsive genes	[92]
*OsTZF2 (OsDOS)*	LOC_Os05g10670	CCCH-type zinc finger TF	Negative	Age, JA		[93]

## Data Availability

Not applicable.
